# Nanomedicine Strategies for Targeting Tumor Stroma

**DOI:** 10.3390/cancers15164145

**Published:** 2023-08-17

**Authors:** Mei-Chi Su, Susheel Kumar Nethi, Pavan Kumar Dhanyamraju, Swayam Prabha

**Affiliations:** 1 Department of Experimental and Clinical Pharmacology, College of Pharmacy, University of Minnesota, Minneapolis, MN 55455, USA; su000055@umn.edu; 2 Nanovaccine Institute, Department of Chemical & Biological Engineering, Iowa State University, Ames, IA 50011, USA; snethi@iastate.edu; 3 Fels Cancer Institute of Personalized Medicine, Lewis-Katz School of Medicine, Temple University, Philadelphia, PA 19140, USA; pavan.kumar.dhanyam.raju@temple.edu; 4 Department of Cancer and Cellular Biology, Lewis Katz School of Medicine, Temple University, Philadelphia, PA 19140, USA; 5 Cancer Signaling and Microenvironment Program, Fox Chase Cancer Center, Temple University, Philadelphia, PA 19111, USA

**Keywords:** tumor microenvironment, extracellular matrix, collagen, fibronectin, hyaluronic acid, cancer-associated fibroblasts, cancer therapy, nanomedicine

## Abstract

**Simple Summary:**

The tumor microenvironment plays an important role in drug resistance and supports/promotes tumorigenesis. Stromal modulators in combination with nanomedicine therapeutics have recently been investigated for reprogramming the tumor microenvironment. Here, we review the major stromal components and recent advances in the use of stroma-targeted therapies for cancer treatment.

**Abstract:**

The tumor stroma, or the microenvironment surrounding solid tumors, can significantly impact the effectiveness of cancer therapies. The tumor microenvironment is characterized by high interstitial pressure, a consequence of leaky vasculature, and dense stroma created by excessive deposition of various macromolecules such as collagen, fibronectin, and hyaluronic acid (HA). In addition, non-cancerous cells such as cancer-associated fibroblasts (CAFs) and the extracellular matrix (ECM) itself can promote tumor growth. In recent years, there has been increased interest in combining standard cancer treatments with stromal-targeting strategies or stromal modulators to improve therapeutic outcomes. Furthermore, the use of nanomedicine, which can improve the delivery and retention of drugs in the tumor, has been proposed to target the stroma. This review focuses on how different stromal components contribute to tumor progression and impede chemotherapeutic delivery. Additionally, this review highlights recent advancements in nanomedicine-based stromal modulation and discusses potential future directions for developing more effective stroma-targeted cancer therapies.

## 1. Introduction

Cancer remains a leading cause of global morbidity and mortality despite advances in targeted therapeutics. The main reason for treatment failures is drug resistance. Genetic factors such as mutations and copy number variations are a key source of resistance but are not the only determinants [1]. Stroma comprises a collection of cells and matrix components that support the delivery of nutrients and the survival of functional cells [2]. In cancer, the stroma plays a crucial role in tumorigenesis [3], tumor growth [4], metastasis [5], and resistance to chemotherapy [6]. Additionally, elevated interstitial fluid pressure (IFP) and dense tumor stroma in the tumor microenvironment (TME) contributes to high transport resistance [7]. Further, overexpression of drug efflux transporters, such as ATP binding cassette (ABC) proteins [8], and the deregulation of drug-associated tumor targets [9,10] allow tumor cells to escape the cytotoxicity of anticancer drugs.

Another important source of drug resistance is tumor heterogeneity, which can arise from both intrinsic and extrinsic factors. Intrinsic factors primarily result from variations among cancer cell populations within a tumor (intra-tumor heterogeneity) or among patients (inter-tumor heterogeneity). These variations can be attributed to the differences in genetic mutations, copy number variants, RNA expression, and protein abundance [11]. Extrinsic factors arise from the changes in the surrounding environment of cancer cells owing to the presence of fibroblasts, vasculature, and extracellular matrix (ECM). Tumor heterogeneity resulting from the combination of intrinsic and/or extrinsic factors hinders targeted therapy and the identification of biomarkers for selecting appropriate patient populations to maximize the therapeutic efficacy of chemo- and immunotherapeutics [12]. TME alterations, such as increased immunosuppression, secretion of growth factors, various paracrine cytokines, and chemokines to modulate the recruitment, infiltration, polarization, and function of immune cells within it further contributes to tumor progression [13]. Furthermore, current traditional anticancer therapies primarily focus on targeting and eliminating cancer cells, often overlooking the role of the surrounding normal cells and matrix components of TME, resulting in marginal progress in clinical outcomes [14]. Therefore, incorporating considerations for tumor heterogeneity into therapeutic approaches holds promise in enhancing the efficacy of current conventional therapies.

**Figure 1:**
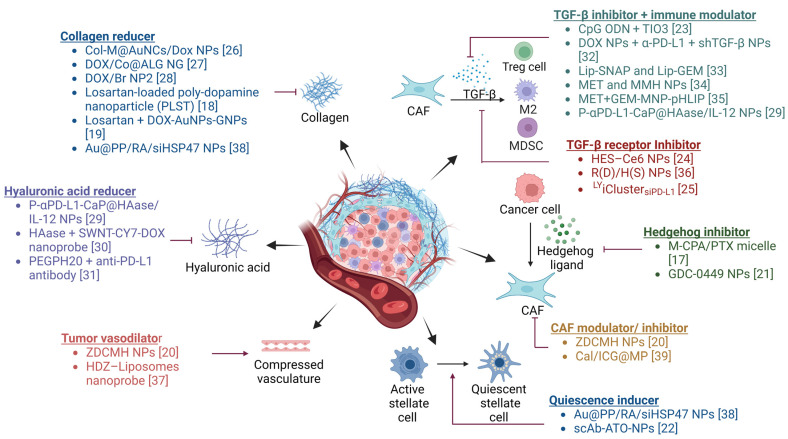
TME modulator combination therapies [17,18,19,20,21,22,23,24,25,26,27,28,29,30,31,32,33,34,35,36,37,38,39]. Various agents that modulate the production of stromal components and/or signaling in the TME have been investigated to overcome resistance to anticancer therapies. Created with BioRender.com. Abbreviations: CAF: cancer-associated fibroblast, Treg: regulatory T cell, M2: M2 phenotype microphage, MDSC: myeloid-derived suppressor cell, Col-M: collagenase-functionalized biomimetic, AuNCs: Au nanocages, DOX: doxorubicin, NP: nanoparticle, Co: collagenase, ALG: alginic acid, NG: nanogel, Br: bromelain, DOX-AuNPs-GNPs: doxorubicin-loaded gold nanoparticles (DOX-AuNPs) onto matrix metalloproteinase-2 (MMP-2)-degradable gelatin nanoparticles (GNPs), Au@PP: mPEG-d-PEI-coated mercaptoundecanoic acid-capped AuNPs, RA: retinoic acid, siHSP47: small interfering collagen-specific molecular chaperone RNA, P-αPD-L1-CaP@HAase/IL-12 NPs: polyethylene glycol shieldable nanodrug P-αPD-L1-calcium phosphate@HAase/IL-12 nanoparticles, HAase: hyaluronidase, SWNT-CY7-DOX: single-walled carbon nanotubes- cyanine7—doxorubicin, PEGPH20: PEGylated human hyaluronidase, ZDCMH NPs: hyaluronic acid gel shell-coated zinc phthalocyanine and bromopentacarbonylmanganese(I) and losartan-coloaded mesoporous silica nanoparticles, HDZ: hydralazine, CpG ODN: cytidine phosphate guanosine (CpG) oligodinucleotide (ODN), shTGF-β: short hairpin RNA-targeting transforming growth factor β, Lip-SNAP: nitric oxide (NO) donor S-nitroso-*N*-acetylpenicillamine (SNAP)-loaded liposomes, Lip-GEM: gemcitabine (GEM)-loaded liposomes, MET: metformin, MMH NPs: MIL-100/mitoxantrone/hyaluronic acid nanoparticles, GEM-MNP-pHLIP: Gemcitabine (GEM) and pH (low) insertion peptide (pHLIP) comodified magnetic nanoparticles, HES-Ce6 NPs: hydroxyethyl starch-chlorin e6 conjugate self-assembled nanoparticles, R(D)/H(S) NPs: doxorubicin loaded rotaxane/SB431542 loaded heparin coated nanoparticles, ^LY^iCluster_si_*_PD-L1_*: LY2157299 and siRNA targeting PD-L1 (siPD-L1) clustered nanoparticle, M-CPA/PTX micelle: cyclopamine (CPA) and paclitaxel (PTX) polymeric micelle, ZDCMH NPs: zinc phthalocyanine (ZnPc, a photosensitizer), bromopentacarbonylmanganese(I) (COMn, a CO donor), and losartan (Dup, a CAF inhibitor) were coloaded inside mesoporous silica nanoparticles (MSNs), Cal/ICG@MPs: tumor cell-derived microparticles co-delivering calcipotriol and Indocyanine green, Au@PP/RA/siHSP47 NPs: all-trans retinoic acid (RA) and siRNA targeting heat shock protein 47 (siHSP47) PEGylated polyethylenimine (PEI)-coated gold nanoparticles, scAb-ATO-NPs: single-chain antibody of fibroblast activation protein-α (scAb_FAP-α_) modified arsenic trioxide (ATO)-loaded nanoparticles. Created with BioRender.com.

Recently, there has been considerable interest in developing anticancer strategies that combine conventional therapies with TME modulators to address drug resistance [15,16]. Figure 1 summarizes different TME modulators currently under investigation. It was found that combination therapies involving TME modulators resulted in higher tumor growth inhibition (TGI) when compared to the groups receiving anticancer drugs without TME modulators. Among the various TME modulators, stromal modulators (e.g., TGF-β inhibitors, hedgehog inhibitors, among others) have demonstrated greater (>70%) tumor inhibition and have advanced to clinical trials for further evaluation [17]. Notably, several U.S. Food and Drug Administration (FDA) approved drugs, including losartan, GDC-0449 (vismodegib), and arsenic trioxide, originally indicated for hypertension, basal cell carcinoma, and refractory or relapsed acute promyelocytic leukemia, respectively, have demonstrated improved anticancer efficacy when delivered in combination with TME modulators [18,19,20,21,22]. Modulation of stroma to overcome immunosuppression can further enhance the efficacy of immunotherapy and other therapeutic modalities such as radiation therapy [23,24,25].

Nanomedicine has been investigated extensively, mainly for applications in cancer drug targeting. Due to the relatively leaky vasculature in tumors compared to healthy tissues, where the range of inter-endothelial cell gaps in tumors is between 200 and 1200 nm [40], nanoparticles in this size range can traverse through these gaps and improve tumor drug accumulation. Nanoparticles including liposomes [41], inorganic nanoparticles [42], polymeric nanoparticles [43], solid lipid nanoparticles [44], micelles [45], inclusion complexes with cyclodextrins [46], and dendrimers [47] have been investigated to deliver chemotherapeutics to tumor tissues, allowing for a reduction in the systemic toxicity. Moreover, because of the progress made in chemical and material sciences, nanoparticles can now be fabricated with high payload capacity and sustained drug release. Nanomedicine can overcome the limitations of conventional approaches such as low bioavailability, low targeted cell membrane penetration, and unintended drug degradation in circulation [48]. Multifunctional nanomedicine systems can be designed using a variety of techniques, including surface targeting ligand modification [49], surface property alteration [50], material composition selection [51,52], physical property optimization [53] and co-delivery of several therapeutic drugs [54,55]. Beyond the field of cancer therapies, the use of nanomedicine in other disease areas, including lung disease [56], cardiovascular disease [57], and bone and cartilage disorders [58], has resulted in improved effectiveness as well. Currently, the FDA and European Medicines Agency (EMA) have authorized over 80 nanomedicine therapeutics [59], and more than 60 clinical studies are ongoing (clinicaltrials.gov, accessed May 2023).

In this review, we first provide an overview of the function of stroma and its role in cancer progression. We focus on three non-cellular components of stroma, collagen, fibronectin (FN), hyaluronic acid (HA), and one key stromal cell type, CAFs. Finally, this review focuses on the use of nanomedicine-based stromal modulation to improve tumor penetration and hence efficacy.

## 2. Stroma: Components and Role in Physiology and Pathology

The stroma is a complex microenvironment that surrounds and supports the proliferation and survival of cells in the body. It is composed of diverse cells, including vascular cells, infiltrating immune cells, and fibroblasts [60]. Vascular cells, such as endothelial cells and pericytes, are responsible for maintaining the integrity and function of blood vessels, which is essential for the transport of nutrients and oxygen to the tissue. Infiltrating immune cells, such as B-cells, T-cells, natural killer (NK) cells, innate lymphoid cells, dendritic cells, and macrophages, play a critical role in generating an immune response against microbial pathogens and malignant cells. Immune cells are attracted to the site of injury or damage through complex cytokine signaling and migrate through the blood vessels to eliminate pathogens and promote tissue repair [61]. Fibroblasts are the major source of ECM components, including collagen, elastin, HA, and other glycosaminoglycans, which play a vital role in maintaining the structural integrity of the stroma [62]. The interactions between these different types of stromal cells are critical for maintaining healthy homeostasis in the microenvironment. However, when homeostasis is disrupted, various diseases such as cancer [63], chronic inflammation [64], and bone marrow disorders with impaired hematopoiesis can occur [65].

The functions of stroma can be divided into two main categories: cytokine network regulation and tissue structure maintenance. Cytokine network regulation controls how cells respond to external and internal stimuli, such as cell damage and healing. For example, when cells are damaged, injured cells initiate the clotting cascade to stop bleeding and begin tissue repair through a series of events, including vasoconstriction, platelet aggregation, and fibrin deposition [66]. These sequential events are controlled by multiple cytokines secreted by stromal cells, including platelet-derived growth factor (PDGF), transforming growth factors α and β (TGF-α and TGF-β), and fibroblast growth factor-2 (FGF-2) [66]. For tissue structure maintenance, fibroblasts increase the stiffness of the stroma by secreting ECM components and soften the stroma matrix by secreting matrix metalloproteinases (MMPs) to break the linkages between ECM components. However, an imbalance in cytokine networks and structure maintenance can lead to many diseases [67]. Keloid scarring is an example of uncontrolled ECM overproduction resulting from collagen types I and III overproduction at the injury site [68,69]. Therefore, a balanced cytokine network and proper tissue structure maintenance are critical for maintaining tissue health and homeostasis [70,71].

## 3. Role of Stroma in Cancer Progression

**Figure 2:**
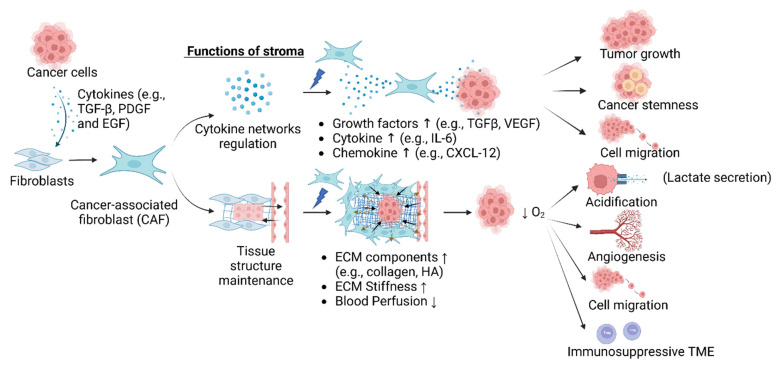
Stroma alteration and its impact on cancer progression. Cancer cells release a variety of cytokines, such as TGF-β, PDGF, and other growth factors, which activate fibroblasts to become CAFs [77]. The CAFs secrete a range of growth factors, cytokines, and chemokines that support tumor growth, induce cancer stemness, and promote migration. Additionally, the overproduction of ECM components by CAFs results in an increase in ECM stiffness and a reduction in blood perfusion. Hypoxia, diminished tissue oxygen levels created by reduced blood flow, leads to a shift in cancer cell metabolism, including upregulation of glycolysis, acidification of the ECM, and initiation of angiogenesis, cell migration, and the formation of an immunosuppressive TME [80,81,82,83]. Abbreviations: TGF-β, transforming growth factor beta; PDGF, platelet-derived growth factor; EGF, epidermal growth factor; CAFs, cancer-associated fibroblasts; VEGF, vascular endothelial growth factor; IL-6, interleukin 6; CXCL-12, C-X-C motif chemokine-12; ECM, extracellular matrix; HA, hyaluronic acid; TME, tumor microenvironment. Created with BioRender.com.

In healthy tissues, the stroma plays a significant role in maintaining tissue structure and function through the regulation of blood vessel formation and the facilitation of immune cell migration and function [72,73,74,75,76]. However, in the presence of cancer, the stroma can be altered and can contribute to tumor growth. CAFs are a phenotype created and activated by tumorigenic signals released by cancer cells [77]. CAFs, in turn, secrete various proteins, including growth factors, cytokines, chemokines, and ECM components, that remodel the tissue structure, promote tumor proliferation, and contribute to therapy resistance [78]. Unlike in normal tissue, where repair processes are tightly regulated, this balance is disrupted in cancer, which is characterized by an overproduction of ECM components and cytokines [79]. This results in a modified stromal environment that supports cancer progression. Figure 2 illustrates how the stroma can contribute to cancer progression.

In the following sections, we introduce how collagen, fibronectin, HA, and CAFs impact cancer progression, respectively.

### 3.1. Collagen

Proliferation, invasion, metastasis, and apoptosis of cancer cells have all been demonstrated to be significantly influenced by collagens, particularly beaded filament type VI and fibrillar collagens [84]. The role of collagen in different types of cancers is shown in Table 1. Collagen type I plays a critical role in the desmoplastic reaction, leading to an increase in fibroblasts and ECM components, and has been shown to contribute to chemoresistance in pancreatic ductal adenocarcinoma (PDAC) [85]. In the desmoplastic reaction, collagen I supports cell proliferation and reduces the sensitivity of tumor cells to anticancer drugs [86]. Previous studies show that sensitivity to 5-fluorouracil (5-FU) was reduced in pancreatic cell lines in the presence of collagen I [85]. Additionally, collagens are also associated with metastasis [87]. Studies have shown that collagen V activated α2β1-integrin and promoted migration of pancreatic cancer cell lines, where higher α2β1-integrin expression increased the likelihood of migration and invasion [88]. Unlike most collagens secreted by myofibroblasts, collagen IV is produced by pancreatic cancer cells and is localized on the cancer cell surface. Upregulation of collagen IV enhances α1β1-integrin and α2β1-integrin binding on the cancer cell surface, promoting PDAC migration and growth [89]. Furthermore, collagens can indirectly impact metastasis through cadherins. Cadherins, which are cell–cell adhesion molecules, are regulated by collagens. The more stable E-cadherin/catenin complex formation between epithelial cells leads to reduced cell migration. Research has found that collagens can disturb the stabilization of the E-cadherin/catenin complex and the presence of collagen types I and III promoted metastasis three- to fivefold in vivo [90].

Type I collagen has been shown to affect metastasis, as exposure to type I collagen results in a more invasive behavior in tumor cells [90]. Barcus et al. demonstrated that the number of circulating tumor cells increased as compared to that in wild-type (wt) mice in an in vivo breast cancer model with accumulated type I collagen distribution. Furthermore, the metastatic lesions were much larger than in the wt mice [100].

Recently, Papanicolaou et al. identified four critical matrisomal clusters via single-cell transcriptomics and proteomics and demonstrated how the structure of collagen I is altered by CAF-secreted collagen XII, resulting in a pro-invasive milieu that supports metastatic spread [101]. Additionally, their studies suggest that collagen XII could be used as a biomarker in breast cancer patients at high risk of metastatic recurrence [101].

Wang et al. demonstrated the role of the interaction between collagen and mutant proto-oncogenes in cancer progression [102,103]. It was shown that silencing of Kras expression significantly reduced collagen I deposition in renal fibrosis, and together with the epithelial–mesenchymal transition (EMT) regulator, SNAIL, mutant Kras increased collagen formation by pancreatic cancer stellate cells (PCAS) [102,103].

Senthebane et al. demonstrated that esophageal squamous cell carcinoma (ESCC) tissues had elevated expression levels of laminins, FN, and collagen when compared to normal tissue [104]. Further decellularization of ECMs abrogated the effect of chemotherapeutics such as epirubicin, 5-fluorouracil, and cisplatin on cell proliferation, cell cycle, and drug-induced apoptosis. Furthermore, the use of chemotherapeutics in combination with anti-fibronectin agents on collagen-deficient ECMs enhanced cancer cells’ susceptibility to chemotherapeutics by 30 to 50% and led to a decrease in tumor invasion and a reduction in the colony formation [104].

### 3.2. Fibronectin (FN)

Multiple studies have shown the critical role played by FN in cancer cell migration, growth, and metastasis as well as drug resistance. The formation of FN networks in CAFs is known to influence the direction of cancer cell migration [105]. Unlike the “mesh-like” FN network produced by normal fibroblasts, CAFs produce a “parallel-like” network that guides cancer cell migration. Studies have also shown that depletion of FN can inhibit the proliferation of PDAC cell lines and increase apoptosis by up to 50% [106]. Additionally, FN has been found to induce chemoresistance by activating the ERK1/2 phosphorylation pathway [107]. Cao et al. showed that FN was overexpressed in gall bladder cancer (GBC) tissues and was linked to poor prognosis in individuals with GBC [108]. Additionally, the authors showed that the GBC-SD and NOZ cells, secreted higher levels of MMP-9 when incubated with recombinant FN. Further, animal studies demonstrated that treatment with FN promoted GBC cell proliferation and metastasis, suggesting the involvement of FN in cell proliferation and invasion via activation of the mTOR signaling cascade during GBC progression [108].

In another study, Von Au et al. demonstrated that circulating FN facilitates the growth of bone metastases by promoting neo-angiogenesis in transgenic mice [109]. Furthermore, they showed that circulating FN enhances its own local production via a positive feedback loop as well as the production of vascular endothelial growth factor (VEGF) that is retained in the matrix [109]. Both FN and VEGF then cooperate in neo-angiogenesis. Additionally, they showed that FN content in tumors correlates with the number of blood vessels and tumor growth in mouse models [109]. Ou and colleagues demonstrated the critical function that FN plays in renal cell cancer (RCC) [110]. In human Caki-1 and RCC 786-O cells, RNA interference (RNAi)-based silencing of FN resulted in decreased cell growth as well as clonogenic cell growth in long-term cultures [110]. In addition, silencing FN in 786-O cells reduced their chemotactic migration toward 10% fetal bovine serum and wound healing relative to the control cells. Further analysis demonstrated reduced expression of vimentin and cyclin D1 as well as TGF-β1 production following FN gene silencing. The administration of exogenous FN and TGF-β1 to the silenced cells reversed the effects on cell proliferation and migration. These cells had elevated levels of TGF- β1, cyclin D1, and vimentin expression [110]. Table 2 summarizes the pathological functions of FN in solid tumors.

### 3.3. Hyaluronic Acid (HA)

High overexpression of HA has been linked to poor prognosis in patients with pancreatic, colorectal, prostate, and ovarian cancer [117,118]. Higher expression of HA has also been shown to be a potential biomarker of metastasis in breast cancer patients [119,120]. HA is produced by HA synthase (HAS1, HAS2, and HAS3) and degraded by hyaluronidases (HYAL1 and HYAL2). It is also a well-known ligand of both a cell surface HA-binding transmembrane glycoprotein (CD44) and a receptor for HA-mediated motility (RHAMM) [121]. CD44 is often overexpressed on the surface of cancer cells and is viewed as a biomarker in many solid tumors [122]. For cell motility, cancer cells secrete hyaluronidases to degrade HA into smaller molecules and cause cleavage of CD44, inducing filopodia formation and promoting cell migration [123]. Importantly, the HA-CD44 interaction is known to activate downstream EGFR-mediated pathways, which promote cancer cell proliferation and migration [124]. A study by Song et al. demonstrated that silencing HAS2 and HAS3 resulted in a reduction in HA synthesis, which subsequently inhibited the proliferation of non-small cell lung cancer cells and reduced CD44, RHAMM, EGFR, Akt, and ERK expression [125]. Recently, HA-CD44 interactions have been utilized in HA-conjugated nanoparticles to improve anticancer efficacy via enhanced internalization of nanoparticles in CD44-overexpressed tumor cells [126,127]. Additionally, CD44 is a well-known stem cell marker and is associated with chemoresistance [128]. A study using athymic nude mice found that mice implanted orthotopically with CD44^high^ pancreatic cancer cell lines were more resistant to gemcitabine compared to those implanted with CD44^low^ pancreatic cancer cell lines [129]. Ricciardelli et al. observed that about 75% of ovarian cancer patients had increased serum HA levels after carboplatin treatment [130]. Similarly, in their in vitro study, HA, HAS2, HAS3, and ABCC2 expression increased after carboplatin treatment [130]. Furthermore, the authors found that different forms of CD44 display distinctive features. Cells with prominent levels of the standard form of CD44 tend to have the EMT phenotype, such as fibroblast-like appearance, high vimentin, and low E-cadherin. Conversely, high levels of CD44 variant isoforms display more epithelial-like phenotypes [129]. Table 3 summarizes the ways in which HA influences tumor progression.

### 3.4. Cancer-Associated Fibroblasts (CAFs)

CAFs are key players in modulating the TME and play a crucial role in the regulation of tumor structure [84,138]. CAFs secrete growth factors and cytokines, such as TGF-β, VEGF, interleukin-6 (IL-6), interleukin-8 (IL-8), CC-chemokine ligand 5 (CCL5), C-X-C motif chemokine ligand (CXCL) 8, and CXCL12, which results in an immunosuppressive environment [139,140,141]. Different cell populations can give rise to CAFs, including endothelial cells, pericytes, adipocytes, stellate cells, tissue-resident fibroblasts, epithelial cells, fibrocytes, and mesenchymal stem cells [142,143]. CAF markers include alpha smooth muscle actin (αSMA), fibroblast activation protein alpha (FAP-α), actin alpha 2 (ACTA2), fibroblast-specific protein 1 (FSP1), and platelet-derived growth factor receptor alpha and/or beta (PDGFRα/β) [139]. The expression level of these markers depends on the origin and surrounding environment [144]. Furthermore, the functions and heterogeneity of CAFs are dependent on the cancer stage, tumor type, and spatial location. For example, breast cancer has four subtypes of CAFs—CAF-S1, CAF-S2, CAF-S3, and CAF-S4—with distinguishable functions. CAF-S1 regulates Treg-mediated immunosuppression and CAF-S4 is involved in cell contractility [145,146]. Based on spatial locations, CAFs in pancreatic cancer can be categorized into myofibroblastic CAFs (My-CAFs), inflammatory CAFs (ICAFs), and antigen presenting CAFs (Ap-CAFs). My-CAFs are located near cancer cells and express high levels of αSMA. In contrast, ICAFs are located farther away from cancer cells, express relatively low levels of αSMA, and secrete high levels of cytokines and chemokines [147]. Ap-CAFs mainly regulate immune response. The main function of My-CAFs is ECM construction and remodeling by ECM components (e.g., collagen, FN, elastin, tenascin) and enzyme secretion (e.g., MMPs) [143]. ICAFs tend to secrete immune-relevant cytokines (e.g., IL-6, IL-11 and leukemia inhibitory factor, CXCL1, and CXCL2), which may promote cancer progression [148], migration [149], and chemoresistance [150]. Ap-CAFs tend to regulate immune response (e.g., CD4+ T-cell deactivation) [151]. Although different phenotypes of CAFs function distinctly, the phenotypes of CAFs can be interchangeable according to their location and biochemical microenvironment [152].

## 4. Mechanisms of Stroma-Mediated Therapy Resistance

### 4.1. Barrier to Tumor Cell Drug Accumulation

The tumor stroma limits the efficacy of chemotherapy through several mechanisms. The high IFP observed in the tumor stroma impedes the convective distribution of chemotherapeutics to cancer cells [153,154]. Further, the tumor ECM is more rigid than normal tissue ECM, which limits the diffusive transport of chemotherapeutics to the site of action [155]. The blood vessels in the tumor tissue are compressed by the rigid tumor ECM, which limits perfusion to tumors, resulting in a reduced drug delivery [156]. Provenzano et al. observed limited penetration of doxorubicin into the tumor due to high IFP. Coadministration of doxorubicin with PEGylated human hyaluronidase (PEGPH20) was found to be effective in degrading stromal HA and normalizing IFP, resulting in a greater than sixfold increase in doxorubicin delivery to tumor cells in pancreatic cancer mouse models [153]. In addition, tumor tissue was less rigid and highly vascularized following treatment with PEGPH20 [153].

The strong binding of the ECM proteins to cancer cells allows cancer cells to escape cytotoxic drugs through a process known as cell-adhesion-mediated drug resistance (CAM-DR) [79]. This CAM-DR is mediated by various adhesion molecules, such as integrins [157], C-X-C motif chemokine receptors (CXCR) [158], and β-catenin [159]. Waldschmidt et al. observed increased cytotoxicity when co-culturing stromal cells and multiple myeloma cells with bortezomib and the CXCR4 inhibitor plerixafor [160]. Ding et al. demonstrated improved tumor inhibition when temozolomide (TMZ) was co-administered with oroxylin A, a β-catenin pathway inhibitor, compared to that with just TMZ in a glioblastoma xenograft mouse model [161]. Moreover, proteins such as periostin secreted by pancreatic stellate cells (PSCs) and stroma have been shown to promote carcinogenesis and resist gemcitabine, a chemotherapy drug commonly used in the treatment of pancreatic cancer [162].

### 4.2. Expression of Factors That Contribute to Chemotherapy Resistance

Chemotherapy alone is often not sufficient to eliminate the entire cancer cell population, as cancer cells can develop resistance mechanisms and receive support from the tumor microenvironment. The stroma can activate a stress response in response to DNA damage caused by chemotherapy, leading to the secretion of growth factors and cytokines that promote tumor cell survival, proliferation, migration, invasion, and metastasis [79]. This creates a complex network of interactions between cancer cells and the stroma that can promote chemoresistance and impede the efficacy of chemotherapy [163].

The stromal cells secrete enzymes (for, e.g., cytochrome P450) that degrade drugs and reduce their therapeutic activity [164]. It has been reported that CAFs secrete growth factors and cytokines leading to drug resistance and relapse in tumors [165]. Amin et al. found that the viability of neuroendocrine (NT-3) cells in response to everolimus treatment was increased by about 27% when cultured in CAF-conditioned media [166]. Lu et al. found that the stiffness of the ECM due to high expression of Calponin-1 in CAFs resulted in 5-FU resistance in gastric tumor cells [167]. Interestingly, a synergistic interaction of CAF-triggered growth factors with hypoxia-inducible factor (HIF-1α) was found to increase chemotherapy resistance and enhance the stemness of cancer stem cells in colorectal cancer [168]. In addition to these, the accumulation of CAFs in tumor tissues can promote drug resistance by inducing the expression of insulin-like growth factor 2 (IGF2) and insulin-like growth factor receptor-1 (IGF-1R) signaling, which leads to P-glycoprotein expression and increased drug efflux [169]. In addition to CAFs, tumor-associated macrophages (TAMs) also contribute to drug resistance through various signaling mechanisms. For instance, 5-FU treatment in gastric cancer promotes the accumulation of reactive oxygen species (ROS) that activates HIF-1α signaling. This, in turn, stimulates the TAMs to produce growth differentiation factor 15 (GDF15) that contributes to drug resistance by enhancing tumor cell fatty acid β-oxidation [170].

### 4.3. Resistance to Radiation Therapy

Radiation therapy, like chemotherapy, can lead to DNA damage and fibrosis in the ECM, resulting in survival and proliferation of stromal cells and resistance of cancer cells to radiotherapy [171]. Additionally, radiation activates CAFs, leading to the promotion of EMT and cancer cell invasiveness while inhibiting the interaction between CAFs and pancreatic cells through the CXCL12/CXCR4 pathway [172]. Studies have shown that ionizing radiation can also trigger an upregulation of integrin expression in fibroblasts, as seen in in vitro and in vivo models, and patient samples [173]. For example, radiation was shown to activate focal adhesion kinase (FAK) and upregulate β1 integrin in PSCs, providing protection to pancreatic cancer cells [174]. In another report, Yao and colleagues demonstrated α5β1 integrin upregulation in pancreatic tumor xenografts (Panc-1, MiaPaCa-2, and BxPC-3) that were exposed to radiation [175]. Moreover, stromal fibroblasts exposed to irradiation develop an ability to secrete chemoresistance-inducing factors [176]. Co-culturing pancreatic cancer cells with irradiated fibroblasts has been shown to significantly enhance cancer cell invasiveness through the activation of the hepatocyte growth factor (HGF)/c-Met signaling pathway [177].

### 4.4. Hypoxia

A key feature of solid tumors is abnormal vasculature, which results in reduced and uneven supply of oxygen [178]. The consequent hypoxia induces further changes in stromal cell biology within the TME that promotes tumor progression and drug resistance [179,180,181]. Stromal cells in the TME undergo several metabolic derangements following activation of hypoxia inducible factor (HIF) and utilize the “Warburg effect” to reduce their dependence on oxygen levels [182]. Cancer cells and CAFs secrete IL-6 to induce HIF-1α expression that modulates downstream genes responsible for chemotherapeutic resistance in cancer cells [183,184]. In addition, HIF contributes to ECM homeostasis, as evidenced by the involvement of HIF-target genes in the synthesis, modification, and degradation of collagen [185,186]. Furthermore, hypoxia tolerant cells have shown to be resistant against most anticancer therapeutics including photodynamic therapy (PDT) [187], immunotherapy (IT) [188], radiotherapy (RT) [189,190], and chemotherapy (CT) [180,191,192]. Hypoxia confers chemotherapy resistance to cancer cells by regulating cell cycle arrest [193], inhibiting senescence and cancer cell apoptosis, and controlling mitochondrial activity, p53, and autophagy [194,195]. Additionally, hypoxia contributes to chemoresistance by upregulating the p-glycoprotein drug efflux pump [196].

## 5. Strategies for Targeting Stromal Interactions

To overcome ECM-associated transport barriers, various strategies have been developed to target signaling pathways associated with the ECM, including inhibition of TGF-β and Hedgehog (Hh) signaling pathways as well as stromal depletion and regulation of CAFs. Additionally, these strategies in combination with nanomedicine provide a more effective way to deliver anticancer drugs while minimizing toxicity to normal cells [197,198].

Nanoparticles formulated using proteins [199,200,201], lipids [202,203,204], and polymers [205,206,207] have been commonly used to encapsulate chemotherapeutic drugs. Several nano-formulations including Abraxane^®^, Genexol-PM, Caelyx, and Onivyde have been approved for treating metastatic cancer [208]. In addition, nanoparticles formulated using inorganic materials such as gold [209], silver [210], and mesoporous silica [211] have demonstrated great potential for implementing novel modalities such as photodynamic and photothermal therapy [212,213,214]. For cancer therapy, use of nano-delivery systems allows cellular toxins [215], radioisotopes [216], and chemotherapeutic agents [217,218] to be targeted to a greater extent to the tumor cells with minimal effects on normal cells. However, these systems rely on passive accumulation in the tumor through the enhanced permeation and retention (EPR) effect and have demonstrated only limited therapeutic benefit in clinical practice [219]. The EPR effect is not observed in all tumors and the targeting effectiveness is low (typically, less than 5% of the administered dose accumulates in the tumor) [220,221]. As discussed in previous sections, greatly elevated IFP and dense tumor stroma are barriers to the uniform distribution of drugs and nanocarriers within the tumor tissue [222,223]. Thus, modulating TME to improve the delivery of nanocarriers has been pursued.

Nanomedicine approaches to normalize the hypoxic regions by the delivery of agents such as nitric oxide [224], HIF inhibitors, and oxygen have been utilized to target hypoxic solid tumors [225]. Perfluorocarbons (PFCs), which are made by combining fluorine and carbon molecules, have high oxygen-carrying potential and high biocompatibility and, therefore, have been used in anti-hypoxic nanotherapeutics [226]. Furthermore, PFCs have oxygen shuttling and donating capabilities similar to hemoglobin (Hb) [226]. Further, nanocarriers have been used to control drug release in the TME by conjugating TME-responsive ligands on the surface of nanocarriers or utilizing TME-responsive linkers [227,228]. In the following sections, we introduce examples of strategies targeting stromal interactions to improve anticancer efficacy.

### 5.1. Targeting Signaling Pathways

#### 5.1.1. TGF-β

In the tumor stroma, TGF-β signaling promotes many cancer progression events, such as activation of CAFs [229], generation of an immunosuppressive environment [230,231], regulation of EMT [232,233,234], angiogenesis [235,236], and reprogramming of cancer metabolism [237]. Therapies that inhibit TGF-β signaling have been shown to significantly improve tumor suppression compared to single therapies. Table 4 summarizes TGF-β-targeted combination strategies.

Pei et al. administered fraxinellone, a TGF-β signaling regulator, in CGKRK-modified nanoparticles (Frax-NP-CGKRK) to evaluate tumor inhibition and found that fraxinellone nanoparticles regulated the TGF-β signaling pathway and were involved in the inactivation of CAFs and M2 macrophage polarization. The combination therapy of fraxinellone nanoparticles and siKras gene resulted in twofold higher tumor inhibition compared to siKras gene therapy alone. Compared to simultaneous combination therapy, sequential combination therapy (fraxinellone nanoparticles followed by siKras gene therapy) resulted in better tumor inhibition and longer median survival [242]. Similar results were reported by Feng et al. where i.v. administration of CAF targeted α-mangostin nanoparticles, followed by acid-triggered triptolide micelles, resulted in a prolonged survival (50 days) and significantly higher tumor inhibition (*p*<0.001) compared to the combination delivery of the chemotherapeutic agent and CAF-targeted agent (38 days) in an orthotopic pancreatic mice model [238].

Another example is all-trans retinoic acid (ATRA), which has been shown to induce PSC quiescence and disable TGF-β activation [243]. Han et al. co-delivered ATRA, gemcitabine, and small interfering RNA targeting heat shock protein 47 (siHSP47) and achieved about a 75% reduction in tumor weight in the Panc-1/PSC subcutaneous mouse model compared to the saline-treated control group [38].

Combination therapies targeting TGF- β and the hypoxic tumor environment have also been investigated using nanomedicine approaches. Liu et al. developed Gd-metallofullerenol (Gd@C82(OH)22) nanoparticles to target both TGF-β and HIF-1α in mice models of triple-negative breast cancer (TNBC) [244]. These nanoparticles demonstrated preferential penetration of TME and delivery of targeted therapeutics to tumor cells only [244].

#### 5.1.2. Hedgehog Signaling

Hedgehog (Hh) signaling has been described as the “Achilles’ heel” in cancer due to its crucial functions, including promoting tumor proliferation, cancer stem cell renewal, and ECM component synthesis [245,246]. Studies have demonstrated that combining a Hh inhibitor with current standard cancer therapy improves efficacy and alters the TME. For example, Zhao et al. co-delivered micelles encapsulating cyclopamine, an Hh inhibitor, and paclitaxel and observed several TME changes, such as hypoxia reduction, vascular normalization, and stroma reshaping in orthotopic PDAC tumor models [17].

Similarly, Wang et al. co-delivered SN-38, the active metabolite of irinotecan, and GDC-0449, an Hh inhibitor, to treat fibroblast-enriched PDAC and observed reductions in multiple fibroblast activation markers, such as α-SMA, collagen, and glioma-associated oncogene transcription factor-1 [21]. Furthermore, other sonic Hh inhibitors, such as vismodegib [247] and NVP-LDE225 [247,248], have shown synergistic effects when combined with chemotherapy or biologics in treating solid tumors. For example, the combination of vismodegib and gemcitabine significantly delayed pancreatic tumor growth compared to gemcitabine monotherapy in animal models. Additionally, the combination of vismodegib with Abraxane^®^ or Doxil^®^ resulted in a better antitumor effect than monotherapy [247].

Another drug shown to target stroma is IPI-926 (Infinity Pharmaceuticals). IPI-926 targets the transmembrane protein Smoothened (Smo), thereby inhibiting the Hh signaling pathway, and has shown therapeutic promise against various Hh-dependent malignancies. For example, mouse models of pancreatic cancer treated with IPI-926 demonstrate a reduction in tumor-associated stromal tissue and an increase in intra-tumoral vessel density [249]. Furthermore, IPI-926 improved the efficacy of simultaneously dosed systemic drugs such as gemcitabine, which led to a decrease in tumor burden and improved survival in mouse tumor models [249].

### 5.2. Stromal Depletion

The overproduction of ECM components in solid tumors increases the stiffness of stroma, which creates a transport barrier for the delivery of drugs and the infiltration of immune cells. To address this issue, antifibrotic agents have been investigated to reduce matrix stiffness and improve the delivery efficiency of chemotherapeutics.

One of the key developments in targeting stroma has been the development of PEGPH20, a recombinant version of the human hyaluronidase enzyme [250,251]. In multiple solid tumors, treatment with PEGPH20 significantly degraded HA, a glycosaminoglycan and a major component of the tumor stroma. For example, patients with HA-high metastatic PDAC experienced a twofold increase in progression-free survival and an improvement in overall survival when given PEGPH20 together with Abraxane^®^ and gemcitabine, according to a phase 2 clinical study [252]. However, the results from a subsequent phase III clinical trial did not show a significant improvement in overall survival in HA-high patients [253]. In another study, the combination of PEGPH20 with Pembrolizumab (Pembro) was shown to be effective in patients with advanced or metastatic non-small cell lung cancer [254]. A study by Morosi et al. in 2021, demonstrated that restructuring the stroma of HA-rich tumors by depleting HA with PEGPH20 pre-treatment is a potentially effective method to increase the therapeutic effectiveness of anticancer medications without increasing their toxicity [255]. Though a number of studies have demonstrated that PEGPH20 may be useful as a therapeutic agent, the thromboembolic consequences of PEGPH20 need to be taken into consideration.

Another approach to improving tumor perfusion and drug delivery is the use of tissue plasminogen activator (tPA). Kirtane et al. used tPA to improve the distribution of Doxil^®^ into the core of the tumor without affecting distribution in normal tissues [256]. Banerjee et al. utilized Minnelide^TM^, a water-soluble analog of triptolide, to reduce stroma components in human-tumor-derived xenografts. This approach resulted in a significant reduction in collagen stabilization, HA synthase expression (*p* < 0.05), CAFs, and tumor cell viability [257]. In another study, Zhu et al. developed polyethylene glycol (PEG), 2-deoxyglucose (DG), and fluorescently tagged CdTe quantum dots (Qds) to deliver siRNA against HIF-1α [258]. These nanocarriers were found to rupture and release their siRNA load in hypoxic TME thereby improving the tumor perfusion and antitumor efficacy [258]. Qds have also been utilized for image-guided delivery of diagnostic and therapeutic agents [258]. PFC@PLGA-red blood cell membrane (RBCM) nanoparticles were used to deliver oxygen to the interior hypoxic regions [259]. Song et al. developed near-infrared light-activated PFC-loaded Bi2Se3 nanoparticles to promote oxygenation and overcome radiotherapy resistance in hypoxic tumors [226].

The use of antifibrotic agents can also lead to a less immunosuppressive tumor microenvironment. Elahi-Gedwillo et al. reported a correlation between the content of HA in the stroma and the infiltration of immune cells. They observed that treatment with halofuginone, an antifibrotic agent, increased immune cell infiltration more than threefold compared to the control group. After 24 h of treatment, CD8+CD3+ cells, CD11b+ cells, and iNOS+ cells increased by about twofold, fivefold, and tenfold, respectively [260]. Another antifibrotic agent, fraxinellone, has also been shown to convert the TME to a less immunosuppressive state by increasing T-cell and NK cell populations, interferon-gamma (IFN-γ) secretion, and decreasing the number of myeloid-derived suppressor cells [92]. These findings suggest that antifibrotic agents may be useful in enhancing the effectiveness of immunotherapy for cancer.

Nanomedicine approaches have been investigated to target the desmoplastic stromal environment and deliver chemotherapeutic agents following the depletion of resistant stromal matrix. Han et al. investigated a two-step strategy to deliver metformin followed by administration of gemcitabine conjugated iron oxide nanoparticles for pancreatic cancer therapy [35]. Metformin administration was found to induce stromal disruption and reduce TGF-β expression that resulted in the suppression of pancreatic stellate cell (PSC) activity. Further, inhibition of PSC desmoplastic activity to form collagen and α-smooth muscle actin resulted in improved delivery of magnetic nanoparticles immobilized with gemcitabine. This two-step approach resulted in greater than 90% inhibition of tumor growth in orthotopic and subcutaneous mouse models of pancreatic cancer. Similarly, Chen et al. investigated a two-step sequential delivery of S-nitroso-*N*-acetylpenicillamine (SNAP), a nitric oxide donor-loaded liposomes (Lip-SNAP), followed by administration of gemcitabine-loaded liposomes in a mouse model of PDAC [33]. Inhibition of desmoplastic stroma production by Lip-SNAP resulted in the enhanced penetration of gemcitabine liposomes and improved therapeutic efficacy. In another study, a mesoporous silica nanoparticle (MSNP) carrier system loaded with gemcitabine and paclitaxel demonstrated improved efficacy of gemcitabine due to the dual effect of paclitaxel in inducing tumor stroma suppression and inhibiting the expression of the GEM-inactivating enzyme cytidine deaminase (CDA) in a PANC-1 orthotopic model [261]. Table 5 summarizes the combination strategies targeted towards depletion of stromal matrix components.

In another study, PEGylated polyethylenimine-coated gold nanoparticles were investigated for TME-responsive delivery of all-trans retinoic acid and siRNA targeting heat shock protein 47 [38]. Gold nanoshells resulted in improved accumulation of chemotherapeutics and improved efficacy, by inducing quiescence of PSCs and inhibiting hyperplasia of the ECM. Recently, Karimnia and colleagues tested in vitro heterocellular 3D co-culture models in combination with imaging and microrheology to investigate the effect of photodynamic stromal depletion in pancreatic cancer [263]. This approach resulted in improved delivery of miR-21-5P (oncomiR)-loaded nanoparticles in pancreatic ductal adenocarcinoma. Mardhian and colleagues [262] demonstrated the efficacy of relaxin-2 conjugated superparamagnetic iron oxide nanoparticles (SPIONs) to reduce fibrosis by modulating the pSmad2 signaling pathway. SPIONs resulted in enhanced tumor cell kill by reducing the expression of stromal components (collagen I, CD31, and desmin) and improving the delivery of gemcitabine [262]. Kang et al. utilized the endohedral metallofullerenol Gd@C82(OH)22 nanoparticles system to inhibit the activity of the matrix metalloproteinases (MMPs) and demonstrated the efficacy of Gd nanoparticles in mouse xenograft models of PDAC [264]. As demonstrated through these multiple studies, nanomedicine strategies have resulted in the improved delivery and penetration of chemotherapeutics in the presence of stromal depleting agents.

### 5.3. Regulation of CAFs

CAFs have gained increased attention in recent years due to their role in regulating tumor–stroma crosstalk [265], producing ECM components [84], and creating an immunosuppressive TME [139]. CAF-like cell models have been created from normal stromal cells such as stellate cells by treating them with TGF-β [266,267]. Drug delivery systems conjugated with CAF-targeting ligands that have high binding affinities to receptors or proteases of CAFs have been investigated to specifically target CAFs. For example, Chen et al. decorated FH-peptide (FHKHKSPALSPVGGG), a tenascin C targeting peptide [268], on navitoclax-loaded nanoliposomes and showed that the system had higher cytotoxicity on CAFs than on tumor cells [267]. Similarly, Guo et al. observed that more FH nanobubbles were located around CAFs compared to non-targeted nanobubbles [266]. Table 6 summarizes different CAF targeting strategies that have been pursued previously.

An alternative strategy for controlling therapeutic release specifically surrounding CAFs is using environment-responsive peptides. FAP-α is a protease secreted specifically by CAFs. Utilizing such a protease allows for active control of drug release since the peptide is mainly cleaved by CAFs. This method has been used to control the release of smaller particles from larger carriers to enhance penetration of particles in solid tumors, known as a size-shrinkage strategy. For example, Yu et al. used liposomes with FAP-α-responsive cleavable amphiphilic peptide (CAP) to control the release of small albumin-paclitaxel complexes (about 7.9 nm diameter) surrounding CAFs, resulting in increased drug penetration in the tumor, from 100 μm to 500–1000 μm [269]. Similarly, Zang et al. utilized FAP-α-responsive CAP in their drug delivery system to control doxorubicin release in the TME. This control release system significantly reduced CAF marker expression, such as α-SMA, FAP-α, and TGF-β levels and further inhibited tumor growth [270]. These results showed that CAF-targeting peptides successfully improved drug penetration due to the depletion of the CAF stromal barrier.

The angiotensin II receptor antagonist losartan was shown to inhibit type I collagen production by CAFs in various desmoplastic models of human breast, pancreatic, and skin cancers in a dose-dependent manner and to enhance the distribution and efficacy of therapeutic nanoparticles [19,273]. However, concerns about cancer recurrence and metastasis resulting from the depletion of CAFs have been observed [274,275]. Therefore, CAF modulation has become an alternative way to avoid the risk of cancer recurrence. For example, Cun et al. utilized 18β-glycyrrhetinic acid (GA) to reduce Wnt16 expression in CAFs located around blood vessels, since Wnt16 causes resistance to chemotherapy and can facilitate metastasis. Utilizing a size-switching strategy controlled by MMP-2, which is present in high concentrations in the tumor microenvironment, the team was able to demonstrate enhanced accumulation of GA-loaded large particles (~88.0 ± 1.1 nm) around blood vessels to suppress Wnt16 expression in CAFs and penetration of gemcitabine conjugated small particles (~28.7 ± 4.1 nm) to deep tumor locations. In vivo antitumor efficacy data showed that this approach resulted in significant tumor growth inhibition compared to controls and no tumor recurrence was observed eight days after the last dose [271].

Tumor cells and CAFs communicate through chemokines and cytokines. CXCL12, a secretory chemokine produced by CAFs, has been shown to play a role in tumor cell invasion [276,277], migration [278], and angiogenesis [279]. To target this communication, Lang et al. developed an siRNA delivery system that downregulates CXCL12 expression in CAFs. This system consisted of an FAP-α antibody for targeting CAFs, a cell-penetrating peptide, and siCXCL12. The delivery system was found to be effective in targeting the CAFs, as evidenced by increased G0/G1 phase arrest of CAFs, inhibition of tumor cell migration, and downregulation of several cytokines involved in cell proliferation, angiogenesis, and cell migration. Additionally, the study found that adding a CAF-targeting ligand significantly improved the gene silencing in CAFs. In vitro studies showed that inclusion of the FAP-α antibody increased inhibition of CXCL12 mRNA expression, α-SMA expression, cell migration, and endothelial cell tube formation compared to that with the non-specific IgG. Furthermore, the number of tumor metastases were significantly decreased by the FAP-α antibody conjugated system compared to that with the negative control siRNA nanocomplex [272]. Overall, these stroma modulation strategies have demonstrated strong antitumor, anti-metastatic, and anti-angiogenic effects and have highlighted the importance of stroma–tumor communication in promoting cancer progression.

When formulating therapies related to CAFs, there are several important factors to consider. First, the order of administration should be considered. For example, studies have shown that administering TGF-β interference therapy prior to antitumor therapeutic agents leads to greater therapeutic efficacy than following co-administration [238,242]. Second, the composition of nanoparticles could be important, as shown by the fact that gold nanoparticles can convert activated CAFs to a quiescent state [280]. Third, the risk of drug tolerance should be considered. For example, high doses (100 mg/kg once to twice daily) of Hh inhibitors have been reported to increase the risk of EMT due to over-ablation of the ECM and CAFs, and long-term use of high doses of Hh inhibitors increases the risk of developing Hh inhibitor tolerance [281,282]. A quantitative modeling approach to determine a suitable drug holiday approach may be a solution to reduce the chance of pharmacodynamic tolerance and to determine the optimal dosing regimen for combination therapy [248].

## 6. Conclusions and Future Directions

It is well established that TME plays a crucial role in cancer progression. Targeting the stroma has shown promising results for improving anticancer efficacy. However, previous studies suggest that stromal ablation can carry potential risks of tumor recurrence and metastasis [274]. One potential solution to mitigate these risks is through stromal modulation or re-education. A deeper understanding of the changes in the TME and the effects of products generated after targeting the stroma can provide insights for improving current strategies. For example, knowledge of the increased LMW-HA promoting tumor angiogenesis after hyaluronidase therapy can inform the use of combination therapies to overcome unwanted effects [283]. Additionally, utilizing single-cell sequencing to decipher the heterogeneity of stromal cell populations [284] and quantitative pharmacological analysis to describe the kinetic relationships between stromal cell populations, ECM reduction/production, and cancer metastasis after stroma targeting treatments can provide a foundation for reducing unwanted effects such as cancer metastasis and angiogenesis. Another area that has not been explored in depth is the potential use of nanomedicine strategies to modulate stroma that supports the growth of metastatic lesions. Recent studies suggest that vascularized organs such as lungs and liver are preconditioned by primary tumors to facilitate the ‘seeding’ of tumor cells in these distant sites [285,286]. Developing strategies to interfere with these interactions could help develop new therapeutic approaches to prevent and/or treat metastases. In conclusion, modulating the TME by targeting the stroma presents an opportunity to treat cancer more effectively.

## Figures and Tables

**Table 1:** Pathological functions of collagen in tumor progression.

Cancer Types	Collagen Types	Pathological Functions of Collagen	References
Pancreatic cancer	I	Cell proliferation	[85]
	II, III, IV, and V	Metastasis	[87,88,89,91]
Breast cancer	IA1	Metastasis	[92]
	XIII	Cancer cell stemness and metabolic reprogramming	[93]
Glioblastoma	I	Cancer cell stemness and adherence	[94]
Lung cancer	VI	Promote lung tumor development through IL-23-mediated lung inflammation	[95]
	XVII	Cancer cell stemness and metabolic reprogramming	[96]
	XVIII	NSCLC progression and poor outcome	[97]
	XXIII	NSCLC recurrence	[98]
Ovarian cancer	XI A1	Chemoresistance and cancer recurrence	[99]

Abbreviations: IL-23: interleukin-23; NSCLC: non-small cell lung cancer.

**Table 2:** Pathological functions of FN in solid tumors.

Cancer Types	Pathological Functions of FN	References
Pancreatic cancer	Aligning FN promoted cancer cell migration	[105]
Breast cancer	FN induced tamoxifen-resistant breast cancer cells through PI-3K/Akt activation	[111]
Cervical cancer	FN regulated MMP-2 and MMP-9 transactivation	[112,113]
Lung cancer	Integrin α5β1/FN interaction promoted cancer cell migration	[114]
	FN type III-1c regulated cancer cell resistance to TNF-related apoptosis inducing ligand through αvβ5 integrin activation	[115]
Prostate Cancer	FN regulated MMP-2 expression and activation	[116]

Abbreviations: FN: fibronectin; MMP-2: matrix metalloproteinase-2; MMP-9: matrix metalloproteinase-9; TNF: tumor necrosis factor.

**Table 3:** Pathological functions of HA in tumor progression.

Cancer Types	Pathological Functions of HA	References
Lung cancer	HA facilitated cell proliferation through CD44-RHAMM and stimulated EGFR/ERK/Akt signaling pathway	[125]
Ovarian cancer	HA induced chemoresistance by increasing ABC transporter expression	[130]
Breast cancer	EMT activated metabolic reprogramming through upregulating production of the HA precursor UDP-glucuronic acid	[131]
	LMW-HA facilitated cancer migration, invasion, and neovascularization	[132,133]
	LMW-HA activated EMT, but HMW-HA inhibited EMT, through CD44-twist signaling pathway	[134]
Glioblastoma	HA facilitated cell invasion through RHAMM pathway	[135]
Pancreatic cancer	HA facilitated cancer migration	[136,137]

Abbreviations: HA: hyaluronic acid; RHAMM: receptor for HA-mediated motility; EGFR: epidermal growth factor receptor; ERK: extracellular-signal-regulated kinase; Akt: protein kinase B; ABC: ATP binding cassette; EMT: epithelial–mesenchymal transition; UDP: uridine diphosphate; LMW: low molecular weight; HMW: high molecular weight; MMP-9: matrix metalloproteinase-9; TNF: tumor necrosis factor.

**Table 4:** TGF-β-Targeted Combination Strategies.

Name	Therapeutics Compounds / Function	Tumor Types	Tumor Targeting Strategy	Nanoparticle Type	Animal Model	Ref.
Chemo-therapy Combination	1. α-mangostin: CAFs inactivator through TGF-β/Smad signaling 2. Triptolide: antitumor agent containing acid-triggering micelles	PDAC	CREKA peptide: fibronectin targeting. CRPPR peptide: Neuropilin-1 receptor- targeting.	Organic (polymeric micelles)	PANC-1-luc/NIH3T3 orthotopic nude mice	[238]
	1. Tranilast: TGF-β inhibitor 2. Doxorubicin 3. Doxil^®^ (PEGylated liposomal doxorubicin)	Triple- negative breast cancer	N/A	Organic (liposomes)	4T1 orthotopic BALB/c mice and E0771 orthotopic C57BL/6 mice	[239]
	1. Gemcitabine 2. All-trans retinoic acid: TGF-β inhibitor and inducer of PSC quiescence 3. Heat shock protein 47 siRNA	Pancreatic cancer	N/A	Inorganic (gold NPs)	1. Panc-1/PSC subcutaneous pancreatic tumor-bearing BALB/c nude mice 2. Panc-1-luci/PSC orthotopic pancreatic tumor-bearing BALB/c nude mice	
Immunotherapy Combination	1. Tranilast: TGF-β inhibitor 2. Anti-CTLA-4 3. Anti-PD-1	Triple-negative breast cancer	N/A	N/A	4T1 orthotopic BALB/c and E0771 orthotopic C57BL/6 mice	[239]
Chemo-Photothermal Combination	1. Telmisartan: TGF-β inhibitor 2. Platinum: photothermal therapy 3. Paclitaxel	Breast cancer	ROS- and GSH-responsive linkage	Organic (gelatin NPs) and inorganic (platinum NPs)	4T1-breast-tumor-bearing Balb/c mice	[240]
Gene therapy	1. Anti-p68 siRNAs 2. Anti-STAT3 siRNAs	Breast cancer/ Colorectal cancer	Hyaluronic acid: CD44-targeting	Organic (polymeric)	4T1-breast-tumor-bearing Balb/c mice and CT26-colorectal-tumor-bearing Balb/c mice	[241]
	1. Fraxinellone: TGF-β inhibitor 2. Mutant KRAS siRNAs	Pancreatic cancer	CGKRK peptide: targeting heparan sulfate proteoglycan	Organic (polymeric NPs and lipoprotein NPs)	Panc-1/NIH3T3 orthotropic pancreatic tumor-bearing nude mice	[242]

Abbreviations: TGF-β: transforming growth factor β; CAF: cancer-associated fibroblast; PDAC: pancreatic ductal adenocarcinoma; PEG: polyethylene glycol; PSC: pancreatic stellate cell; NP: nanoparticle; CTLA4: cytotoxic T-lymphocyte-associated antigen-4; PD-1: programmed cell death-1; ROS: reactive oxygen species; GSH: glutathione.

**Table 5:** Stromal-Matrix-Targeted Strategies.

Name	Therapeutic Compounds/Function	Tumor Types	Targeting Strategy	Nanoparticle Type	Animal Model	Ref.
Chemotherapy Combination	1. Gemcitabine 2. Metformin: PSC activation inhibitor	Pancreatic cancer	pH (low) insertion peptide: increasing transmembrane ability in acidic TME	Inorganic (iron oxide NPs)	1. Panc-1/PSC subcutaneous pancreatic tumor-bearing BALB/c nude mice 2. Panc-1-luci/PSC orthotopic pancreatic tumor-bearing BALB/c nude mice	[35]
	1. Gemcitabine 2. S-nitroso- N -acetylpenicillamin: NO donor	Pancreatic cancer	N/A	Organic (liposomes)	1. PSC/PC subcutaneous pancreatic tumor-bearing BALB/c nude mice. 2. PSC/PC-luci orthotopic pancreatic tumor-bearing BALB/c nude mice.	[33]
	1. Gemcitabine 2. Human relaxin-2: PSC activation inhibitor	Pancreatic cancer	N/A	Inorganic (superparamagnetic iron oxide NPs)	Panc-1/hPSC subcutaneous pancreatic tumor-bearing CB17 SCID mice.	[262]
Gene therapy Combination	1. miR-21-5P inhibitor 2. Photodynamic therapy: photodynamic stromal depletion	Pancreatic cancer	N/A	Organic (peptide-based NPs)	N/A	[263]

Abbreviations: PSC: pancreatic stellate cell; hPSC: human pancreatic stellate cell; NP: nanoparticle.

**Table 6:** CAF-Targeting Strategies.

Name	Therapeutic Compounds/Function	Tumor Types	Targeting Strategy	Nanoparticle Type	Animal Model	Ref.
TME modulator	Navitoclax: CAFs apoptotic agent	Hepatocellular carcinoma	Tenascin C targeting peptide, FH, FHKHKSPALSPVGGG.	Organic (liposomes)	Hep G2 tumor-bearing nude mice model	[267]
Chemo phototherapy	(1) Photothermal therapy: IR-780 iodide (Paclitaxel)	PDAC	FAP-α responsive cleavable amphiphilic peptide	Organic (lipid-albumin NPs)	Pan 02 orthotopic tumor mouse models	[269]
Chemotherapy	Doxorubicin	Prostate cancer	(1) FAP-α responsive cleavable peptide(2) Redox-responsive disulfide linkage	Organic (dendrimers)	Co-inoculated with CAFs and PC-3 tumor-bearing nude mice model	[270]
Chemotherapy	(1) 18β-glycyrrhetinic acid (GA): Wnt 16 suppressor(2) Gemcitabine	Breast and pancreatic cancers	MMP-2 responsive peptide, EGPLGVRGK.	Organic (polymeric NPs)	1. Co-inoculated with NIH3T3 and Pan02 pancreatic tumor mice model. 2. Co-inoculated with NIH3T3 and 4T1 cells breast tumor mice model.	[271]
Gene therapy	siCXCL12	Prostate cancer	Anti-FAP-α mAb	Organic (peptide NPs)	Co-inoculated with CAFs and PC-3 orthotopic prostate tumor mice model	[272]

Abbreviations: CAF: cancer-associated fibroblasts; IR: infrared radiation; PDAC: pancreatic ductal adenocarcinoma; FAP-α: fibroblast activation protein alpha; MMP-2: matrix metalloproteinase-2; CXCL12: C-X-C motif chemokine 12.
